# Fatty acid profiles and desaturase-encoding genes are different in thermo- and psychrotolerant strains of the *Bacillus cereus* Group

**DOI:** 10.1186/s13104-015-1288-4

**Published:** 2015-07-31

**Authors:** Sara Esther Diomandé, Marie-Hélène Guinebretière, Benoit De Sarrau, Christophe Nguyen-the, Véronique Broussolle, Julien Brillard

**Affiliations:** INRA, UMR408 Sécurité et Qualité des Produits d’Origine Végétale, 84000 Avignon, France; Université d’Avignon, UMR408 Sécurité et Qualité des Produits d’Origine Végétale, 84000 Avignon, France; INRA, UMR 1333 DGIMI, Université Montpellier, 34095 Montpellier Cedex 5, France; Xurian Environnement, ZAE Béziers Ouest, rue du Jéroboam, 34500 Béziers, France; INRA, UMR408 SQPOV, Site Agroparcs, 228 route de l’Aérodrome, CS40509, 84914 Avignon Cedex 9, France

**Keywords:** *B. cereus* Group, Fatty acids, Desaturases, Temperature tolerance

## Abstract

**Background:**

The *Bacillus cereus* Group consists of closely-related bacteria, including pathogenic or harmless strains, and whose species can be positioned along the seven phylogenetic groups of Guinebretière et al. (I–VII). They exhibit different growth-temperature ranges, through thermotolerant to psychrotolerant thermotypes. Among these, *B. cytotoxicus* is an atypical thermotolerant and food-poisoning agent affiliated to group VII whose thermotolerance contrasts with the mesophilic and psychrotolerant thermotypes associated to the remaining groups I–VI. To understand the role of fatty acid (FA) composition in these variable thermotypes (i.e. growth behavior vs temperatures), we report specific features differentiating the FA pattern of *B.* *cytotoxicus* (group VII) from its counterparts (groups I–VI).

**Findings:**

The FA pattern of thermotolerant group VII (*B.* *cytotoxicus*) displayed several specific features. Most notably, we identified a high ratio of the branched-chain FAs iso-C15/iso-C13 (i15/i13) and the absence of the unsaturated FA (UFA) C16:1(5) consistent with the absence of ∆5 desaturase DesA. Conversely, phylogenetic groups II–VI were characterized by lower i15/i13 ratios and variable proportions of C16:1(5) depending on thermotype, and presence of the DesA desaturase. In mesophilic group I, thermotype seemed to be related to an atypically high amount of C16:1(10) that may involve ∆10 desaturase DesB.

**Conclusion:**

The levels of i15/i13 ratio, C16:1(5) and C16:1(10) UFAs were related to growth temperature variations recorded between thermotypes and/or phylogenetic groups. These FA are likely to play a role in membrane fluidity and may account for the differences in temperature tolerance observed in *B.* *cereus* Group strains.

**Electronic supplementary material:**

The online version of this article (doi:10.1186/s13104-015-1288-4) contains supplementary material, which is available to authorized users.

## Background

In bacteria, the fatty acid (FA) composition of the cell membrane varies according to environmental conditions, as it is involved in bacterial adaptation to environmental changes such as temperature, pressure, and O_2_ availability [1–4]. Among these changes, the effect of temperature on bacterial FA composition is a prominent focus of research. Desaturases responsible for producing unsaturated FAs (UFAs) have been shown to play a role in low-temperature adaptation. The effect of incorporation of low-melting-point UFAs is to maintain membrane fluidity under the physical stress due to cold [5–9]. The number of desaturases varies depending on species, e.g. two desaturases have been identified in *Bacillus cereusstricto* or *ss* [10] whereas *B. subtilis* counts only one known desaturase [11]. The FA composition of bacterial cells is also known to vary according to species, especially in *Bacillus* and related genera [12, 13], and has been included among important features for describing new taxa of the aerobic endospore-forming bacteria [14].

The *Bacillus cereus* Group (*B.* *cereus* *sensu lato or sl*) includes bacterial strains with a wide range of growth temperatures. These strains can be classified by growth-temperature range, from psychrotrophic to thermotolerant strains [15]. These ranges of growth temperatures fit with the seven major phylogenetic groups (I–VII) established by Guinebretière et al. [15, 16] in the *B.* *cereus* Group (see Table 1). This 7-macrogroup classification is the most complete phylogenetic description of the *B.* *cereus* Group and is coherent with all MLST, AFLP, MLEE and genomic data produced since 2004 in the literature [17]. It offers a unique setting to investigate the relation between temperature adaptation and hypothetical factors and can be used to resolve problems tied to effective species delimitation in the Group. Indeed, *B.* *cereus sl* contains seven closely-related species that, although not all genomospecies, can be clearly positioned by reference to each other in the classification of Guinebretière et al. (Additional file 1). Some of them have been described on the basis of singular phenotypic or pathologic traits such as rhizoidal colonies (*B. mycoides*), psychrotolerance (*B. weihenstephanensis*), insecticidal properties (*B. thuringiensis*) [18], enterotoxins (*B.* *cereus* sensu *ss*) [19] and anthrax (*B. anthracis*) [20]. Only two are true genomospecies: the harmless species *B. pseudomycoides* (mesophilic group I) and the pathogenic species *B. cytotoxicus* (thermotolerant group VII) [21], both of which thus share a homogenous growth-temperature range. *B. mycoides*/*B. weihenstephanensis* (psychrotolerant group VI), and *B. anthacis* (a clonal lineage within the highly mesophilic group III) also have a homogenous growth-temperature range as they belong each to a unique phylogenetic group, whereas *B.* *cereus ss* and *B. thuringiensis* represent highly heterogeneous thermotypes across phylogenetic groups II–VI (Additional file 1) [15, 16].

**Table 1:** Distribution of key fatty acids (FA), *desA* and *desB* according to phylogenetic group in *B. cereus sl*

Categories to which the tested strains and the tested genomes belong			FA%										FA profile category based on i13/i15 ratio	% of genomes positive for Δ5 and Δ10 desaturases (TblastN)	
Phylo-genetic group	Growth range (T, °C)	Major thermotype	Saturated FA (SFA)			Unsaturated FA (UFA)								*desA* (Δ*5*)	*desB* (Δ*10*)
			i13	i15	n16	i16:1 (5)*	C16:1 (5)*	i17:1 (5)*	i16:1 (10)^#^	C16:1 (10)^#^	i17:1 (10)^#^	a17:1 (10)^#^			
VII	20–52	T	5.58 ± 1.17	37.95 ± 1.64	11.28 ± 2.14	0.05 ± 0.05	0.02 ± 0.01	0.08 ± 0.03	1.01 ± 0.43	4.09 ± 0.98	1.06 ± 0.47	0.72 ± 0.30	4	0	100
I	10–43	M	11.98 ± 0.49	13.11 ± 0.16	8.69 ± 0.24	0.07 ± 0.05	0.05 ± 0.01	0.13 ± 0.02	3.08 ± 0.17	13.14 ± 0.77	2.54 ± 0.19	1.16 ± 0.08	2	0	100
III	15–45	HM	16.33 ± 0.92	25.69 ± 0.78	7.53 ± 3.23	0.45 ± 0.09	0.31 ± 0.02	6.28 ± 3.18	1.93 ± 0.82	5.76 ± 0.6	3.27 ± 1.55	0.60 ± 0.39	3	99	100
IV	10–45	M	18.66 ± 1.44	20.86 ± 0.59	12.47 ± 0.15	0.26 ± 0.05	0.50 ± 0.03	3.21 ± 0.49	0.81 ± 0.14	6.25 ± 0.34	1.29 ± 0.23	0.54 ± 0.05	2	96	100
V	9–40	IM	18.71 ± 1.34	17.01 ± 1.92	14.55 ± 1.96	0.42 ± 0.13	0.66 ± 0.06	3.93 ± 1.05	0.69 ± 0.22	4.31 ± 1.13	0.79 ± 0.27	0.41 ± 0.13	2	100	100
II	7–40	LP	17.00 ± 1.51	18.94 ± 2.36	14.75 ± 1.55	0.53 ± 0.13	1.24 ± 0.05	5.26 ± 0.5	0.54 ± 0.13	3.50 ± 0.65	0.70 ± 0.09	0.21 ± 0.09	2	100	100
VI	5–37	HP	20.15 ± 3.3	12.02 ± 0.94	14.67 ± 3.23	0.53 ± 0.31	1.09 ± 0.13	8.05 ± 5.34	0.57 ± 0.4	4.99 ± 1.22	1.02 ± 0.61	0.46 ± 0.16	1	100	100

Phylogenetic groups and growth temperature ranges are described in previous works [15, 21].

FA proportions obtained from the FAMEs extractions and GC–MS analysis for a total of 21 representative strains are presented as mean value ± standard deviation (sd) (n = 2–6 representative strains, with 1–3 replicates). FA nomenclature is: iX: iso-FA with X carbons; aX: anteiso-FA with X carbons; nX: saturated FA with X carbons. For unsaturated FA, symbol “:” is prefixed to the number of unsaturations in FA chain; position of unsaturation in FA chain is indicated between parentheses. The symbol “*” indicates that the FA is unsaturated by DesA and “#” indicates that the FA is unsaturated by DesB.

TblastN search was performed to find orthologs of D5 desaturase gene (desA) and D10 desaturase gene (desB) (locus BC_2983 and BC_0400 respectively in strain ATCC 14579T) among 210 B. cereus sl genomes representative of the seven major phylogenetic groups. For both the in silico search of desA and desB orthologs, the number of tested genomes (found in databanks in September of 2014) was 3 for group VII, 74 for group III, 70 for group VI, 7 for group I, 18 for group V, 8 for group II, 30 for group VI.

Thermotypes: *T* thermotolerant, *HM* highly mesophilic, *M* mesophilic, *LP* low psychrotolerant, *HP* highly psychrotolerant.

*Bacillus* *cytotoxicus*, though relatively rare, is known as one of the most virulent pathogenic species of *B.* *cereus* *sl* [16, 21]. Its pathogenicity is mainly attributed to the greater expression and cytotoxic activity of the pore-forming cytotoxin K1 (CytK-1) [22–25], a variant of the cytotoxin K found in many strains of *B.* *cereus* *sl* [16]. *Bacillus* *cytotoxicus* comprises solely thermotolerant strains [15, 21]. In addition to all these particularities, *B.* *cytotoxicus* has been described as displaying a specific FA composition in *B.* *cereus* *sl* [21]. However, this specific FA composition of *B. cytotoxicus* has actually only been validated with reference to 4 of the 7 phylogenetic groups, and the relation between FA composition and the various thermotypes found in *B.* *cereus* *sl* has not yet been studied.

The aim of this study was to determine to what extent the FA composition of *B. cytotoxicus* (phylogenetic group VII) differs from that of all the other phylogenetic groups of the *B.* *cereus* Group, and to what extent these differences relate to the growth-temperature ranges of the groups. In addition, as desaturases are known to play a role in low-temperature adaptation through FA composition and membrane fluidity, we also investigated the presence of desaturase-encoding genes among the available *B.* *cereus* *sl* genomes and the existence of a putative relation between their presence and the FA composition of strains exhibiting different thermotypes.

## Methods

### Strains

The studied strains are listed in Additional file 2 and were representative of all seven phylogenetic groups. Minimal growth temperature (T_min_) and maximal growth temperature (T_max_) were used to determine growth-temperature range (T_min_–T_max_). Their group affiliation and growth-temperature range were determined in previous studies in 2008 and 2013 [15, 21]. As a rule, each phylogenetic group has its own growth-temperature range [15], as presented in Table 1. In the previous work of 2008, T_min_ and T_max_ were determined using a standard test described in the Bergey’s Manual [26], with temperature fluctuation of the incubators being not greater than ±0.2°C for all tested temperatures.

All these strains are referenced in the previous works of 2008 and 2013 [15, 21].These strains have since been conserved in our Laboratory (UMR408 Collection) and the original sources are presented in Additional file 2.

### Thermotypes

The thermotypes were determined from the growth-temperature ranges (T_min_–T_max_) as the resulting phenotype, and are presented in Table 1 by reference to growth-temperature range.

### FA profiling

The FA methyl esters (FAMEs) were extracted using the standardized MIDI protocol (http://www.microbialid.com/PDF/TechNote_101.pdf). Bacterial cells were obtained from culturing at 30°C (at or close to optimal conditions for all seven phylogenetic groups) on trypticase soy broth agar (TSBA, 30 g trypticase soy broth and 15 g Bacto agar; l^−1^) for 24 h as previously described [21]. After extraction, FAMEs were analyzed by gas chromatography-mass spectrometry (GC–MS) (Shimadzu QP2010 system), as previously described [27].

### In silico analysis

A total of 210 *B.* *cereus* *sl* genomes available in databases at the time of the search were used for this study. Genome affiliations to the phylogenetic groups were established previously [28], as described in [16], using *panC* sequence similarity.

Two desaturases have been described in *B.* *cereus*, i.e. DesA and DesB [10], which are responsible for two different types of unsaturation. The DesA enzyme adds a double bond in the ∆5-position of a saturated FA (SFA) while DesB creates an unsaturation in the ∆10-position of an SFA. The presence of each desaturase-encoding gene was thus investigated among all the available *B.* *cereus* *sl* genomes. The search for *desA* and *desB* orthologs with reference to locus_tag *BC_2983* and *BC_0400*, respectively, in the ATCC 14579T genome (i.e. sequence loci described for desaturases in the ATCC 14579 genome) was performed via the Integrated Microbial Genomes (IMG) interface [29]. No other ∆5 or ∆10 desaturase-encoding genes have been reported in the *B.* *cereus* Group. First, candidate homologs were identified based on BLASTp similarities with a 1e-2 E-value cutoff and with low-complexity soft masking (-F’m S’) turned on. Second, orthologous relationships between *BC_2983* or *BC_0400* genes and their respective homologous genes in all other genomes were established through bidirectional best blast hits.

## Results and discussion

### FA composition in *B.* *cereus* sensu lato: general features

The *B.* *cereus* Group displays a specific overall FA pattern setting it apart from the other species of the *Bacillus* genus [21, 30], with short-chain branched FAs (12C and 13C) and a characteristic predominance of iso-C13:0 (Additional file 2). Whatever the phylogenetic group analyzed, three major FAs were identified: iso-C15:0 (i15), iso-C13:0 (i13) and C16:0 (n16). For better visibility, only these three major SFAs and the 7 UFAs (previously linked to cold adaptation [3, 27]) are listed in Table 1. While the n16 SFA did not range widely according to thermotype, the proportion of the other two SFAs (i13 and i15) varied for the most sharply-contrasting thermotypes, i.e. the thermotolerant type (group VII, *B. cytotoxicus*), the highly mesophilic type (group III), and the highly psychrotolerant type (group VI). The SFA i15 accounted for more than 1/13.3 of total FAs in group VII (*B. cytotoxicus*) and thus constituted a marker for this group.

### i15/i13 ratio as a rough indicator of thermotype

**Fig. 1:**
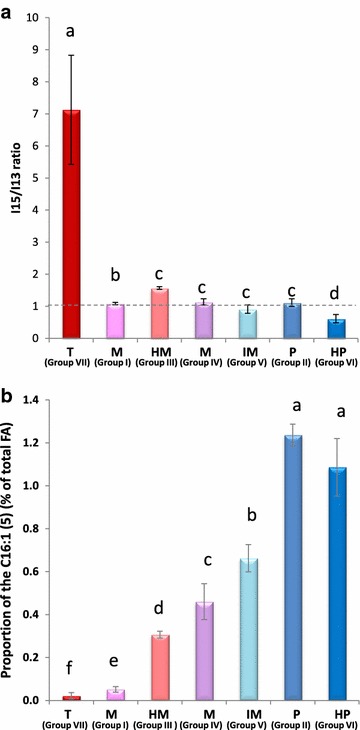
i15/i13 ratio (**a**) and proportion of C16:1(5) (**b**) according to phylogenetic group in *B.* *cereus* *sl.* *Different colors flag* the different phylogenetic groups of *B.* *cereus* *sl. Different letters* above each bar flag significantly different mean values (Student’s *t* test, *p* < 0.05). *T* thermotolerant, *HM* highly mesophilic, *M* mesophilic, *IM* intermediate between mesophilic and psychrotolerant, *LP* low psychrotolerant, *HP* highly psychrotolerant.

We calculated the i15/i13 ratio, defined as proportion of iso-C15:0 divided by proportion of iso-C13:0, for strains belonging to each phylogenetic group (Fig. 1a). i15/i13 ratio was specifically very high in the thermotolerant strains of group VII (7.13 ± 1.7, Fig. 1a) but comparatively very low in the other groups (I–VI), with some variations: largely above 1 (1.57 ± 0.04) for the highly mesophilic strains of group III, largely below 1 (0.61 ± 0.13) for the highly psychrotolerant strains of group VI, and close to 1 in the remaining strains (groups I, II, V and IV). Taken together, these data strongly suggest that the i15/i13 ratio criterion can roughly discriminate *B.* *cereus* *sl* strains into 4 categories (Table 1): (1) highly psychrotolerant strains (group VI), (2) low psychrotolerant to mesophilic strains (groups II, V, IV, I), (3) highly mesophilic strains (group III), and (4) thermotolerant strains (group VII, *B. cytotoxicus*).

### Presence of ∆5/∆10-desaturases and proportion of C16:1(5) as an accurate indicator of thermotype in the most recent branch of *B.* *cereus* *sl*

UFAs unsaturated at the ∆5 position were present in the most recent phylogenetic groups (II–VI, see Additional file 1) yet absent from thermotolerant group VII (*B. cytotoxicus*) and mesophilic group I (Table 1), particularly C16:1(5). We therefore searched for the presence of the genes encoding for the desaturases responsible for the synthesis of UFAs unsaturated at the ∆5 or ∆10 location (*desA* and *desB* respectively) in this group of bacteria. The results indicated that, contrary to *desB*, orthologs of *desA* were not found in all *B.* *cereus* *sl* genomes (Table 1): none of the genomes in groups I and VII contained an ortholog of *desA* gene. The 2.5–4% of negative genomes in groups III and IV may be due to information missing from draft genomes. However, in groups I (*B.* *pseudomycoides* strains) and VII (*B. cytotoxicus* strains), the ortholog of *desA* was truly absent, consistent with the near-zero concentration of ∆5 UFAs observed in groups I and VII.

Considering only groups II to VI, C16:1(5) proportion increased with thermotype, from the highly mesophilic to the highly psychrotolerant groups (with no significant difference between the two psychrotolerant groups II and VI) (Fig. 1b). Despite being produced in low amounts, C16:1(5) proportion appeared a good parameter to discriminate psychrotolerant thermotypes (groups II, VI) from other thermotypes and even to discriminate between mesophilic thermotypes (groups III, IV, V), including those that were not discriminated by i15/i13 ratio (groups II, V, IV).

Atypically in group-I strains, UFAs unsaturated at the ∆10 location were produced in higher proportions than in the other groups (Table 1), particularly C16:1(10), offsetting the lack of UFAs unsaturated at the ∆5 location. This difference presumably contributes to membrane fluidity and allows group I (*B. pseudomycoides*) to exhibit a mesophilic thermotype. In contrast to mesophilic group I (*B.* *pseudomycoides*), the thermotolerant group VII (*B. cytotoxicus*) seems unable to offset this deficiency by producing larger amounts of UFAs unsaturated at the ∆10 location.

### FA composition and putative link with cold adaptation in the *B.* *cereus* Group

Our study highlighted the relation between i15/i13 ratio and *B.* *cereus* Group thermotypes. Another study reported that proportion of i13 was strongly reduced in a *B.* *cereus* ATCC 14579 mutant displaying growth impairment at low temperature compared with its parental strain during growth at low temperature [27]. The i15/i13 ratio recalculated from these data [27] was much higher in the mutant than in the parental strain, emphasizing the putative role of a low i15/i13 ratio for psychrotolerance ability.

The C16:1(5) UFA presumably appeared with ∆5 desaturase DesA in the most recent branch of the phylogeny in *B.* *cereus* *sl* containing groups II–VI (see Additional file 1). Its absence in phylogenetic groups I and VII indicates a link with the whole evolution of the *B.* *cereus* Group. Indeed, groups I and VII belong to two other independent branches at the base of the phylogenetic tree (see Additional file 1). Taken together, these results converge towards a differential process of evolution involving ∆5 UFAs for groups II–VI and ∆10 UFAs for group I. As group VII is basal to the evolutionary tree, followed by group I and then the remaining groups, we can posit that the ancestor of the *B.* *cereus* Group was devoid of ∆5 UFAs and went through adaptation in a few steps. The first step would involve an increase of ∆10 UFAs through group I. The second step would involve ∆5 UFAs through groups II–VI, with a more efficient adaptation from mesophily to psychrotolerance. This is also consistent with the absence of ∆5 desaturase (DesA) in groups I and VII. Interestingly, the same kind of configuration (absence in groups I and VII) was also observed for the two-component system CasK/R, which was recently described as playing a role in *B.* *cereus*-Group cold adaptation [28]. Through this observation, we can also posit that the lack of key genes such as *casK/R* and *desA* might be related to the inability of *B. cytotoxicus* strains to grow at temperatures below 20°C [21], and that these genes probably took part in a more complex mechanism of adaptation in the most recent branch of the phylogeny.

## Conclusion

A link was established between the FA pattern of *B.* *cereus* *sl* strains and ability or inability to grow at low temperature. The FA profile of *B.* *cytotoxicus* (group VII) is highly specific compared to that of the phylogenetic groups I–VI and is relatable to its atypical thermotolerance: high i15/i13 ratio, absence of UFAs unsaturated at the ∆5 location (particularly C16:1(5)), absence of a ∆5 desaturase (DesA). In contrast, the presence of ∆5 desaturase DesA and subtle amounts of C16:1(5) seem to be associated with an advanced mechanism of adaptation, resulting in a large panel of thermotypes through groups II–VI (from mesophily to psychrotolerance). Mesophilic strains of Group I seem to exhibit a specific intermediate state of evolution involving a fairly atypical amount of the ∆10-desaturated C16:1(10).

## Availability of supporting data

The datasets supporting the results of this article are included in Additional file 2.

## Additional files

Additional file 1: Phylogeny of the *B.* *cereus* Group based on *rrs* and ITS1 gene sequence analysis, and position of species in this phylogeny. This file illustrates the position of the *B.* *cereus* *sl* species over 7 major phylogenetic groups according to the classification scheme given in Guinebretière et al. 2008. It also shows the three main branches of evolution in the *B.* *cereus* Group. Additional file 2: Fatty acid (FA) composition of *B.* *cereus* *sl* strains belonging to the 7 phylogenetic groups. This file contains the raw FA composition data of the *B.* *cereus* *sl* strains used to generate Table 1 and Figure 1.

## Abbreviations

ΔX desaturase: a desaturase adding a double bond on the Xth carbon–carbon position of the fatty acid, counting from the carboxyl acid end
aX: anteiso-branched fatty acid with x number of carbons
Cx: y (z): fatty acid with x number of carbons and y number of unsaturation(s) at location z
CytK1: cytotoxin K1
FA: fatty acid
FAME: fatty acid methyl ester
HM: highly mesophilic
HP: highly psychrotolerant
IM: intermediate between mesophilic and psychrotolerant
i15: iso-C15:0
i13: iso-C13:0
iX: iso-branched fatty acid with x number of carbons
M: mesophilic
n16: C16:0
nX: saturated fatty acid with x number of carbons
P: psychrotolerant
sd: standard deviation
*ss*: *sensu stricto*
*sl*: *sensu lato*
T: thermotolerant
UFA: unsaturated fatty acid
xC: x carbon molecule(s)
