# Supplementary material for: Fatty acid profiles and desaturase-encoding genes are different in thermo- and psychrotolerant strains of the Bacillus cereus Group
Source: BMC Res Notes. 2015 Jul 31;8:329. doi: 10.1186/s13104-015-1288-4 (PMC4521489; doi:10.1186/s13104-015-1288-4)
Supplement: Additional file 1: — Phylogeny of the B. cereus Group based on rrs and ITS1 gene sequence analysis, and position of species in this phylogeny. This file illustrates the position of the B. cereus sl species over 7 major phylogenetic groups according to the classification scheme given in Guinebretière et al. 2008. It also shows the three main branches of evolution in the B. cereus Group. [file 13104_2015_1288_MOESM1_ESM.docx]

*B.*

*subtilis*

AF549498

**group VII**

-

INRA AF2

-

**T**

**group VII**

-

NVH 391

-

98

-

**T**

**group VII**

-

NVH 880

-

00

-

**T**

94

**group I**

-

DSM 12442

-

**M**

**group I**

-

CIP 52.59

-

**M**

**group III**

-

CEB 95

-

0033

-

**HM**

**group III**

-

INRA C43

-

**HM**

**group IV**

-

IEBC T04001

-

**M**

**group IV**

-

IEBC T12001

-

**M**

**group V**

-

IEBC T24001

-

**IM**

**group II**

-

IEBC T61001

-

95

78

83

**group VI**

-

SDA NFMO448

-

**HP**

**group VI**

-

WSBC 10377

-

**HP**

**100**

**100**

**51**

**100**

**99**

0.01

*B.*

*subtilis*

AF549498

**group VII**

-

INRA AF2

-

**group VII**

-

NVH 391

-

98

-

**group VII**

-

NVH 880

-

00

-

**group I**

-

DSM 12442

-

**M**

**group I**

-

CIP 52.59

-

**M**

**group III**

-

CEB 95

-

0033

-

**group III**

-

INRA C43

-

**group IV**

-

IEBC T04001

-

**M**

**group IV**

-

IEBC T12001

-

**M**

**group V**

-

IEBC T24001

-

**group II**

-

IEBC T61001

-

**group VI**

-

SDA NFMO448

-

**group VI**

-

WSBC 10377

-

0.01

0.01

**LP**

***B. weihenstephanensis***

***B. mycoides***

***B. thuringiensis* VI**

*B. thuringiensis* II

*B. cereus* II

# **B. pseudomycoides**

*B. thuringiensis* V

*B. cereus* V

***B. thuringiensis* IV**

***B. cereus* IV**

***B. thuringiensis* III**

***B. cereus* III**

***B. anthracis***

***B. cytotoxicus***

**Additional File 1** **Phylogeny of the *B. cereus* Group based on *rrs* and ITS1 genes sequence analysis, and position of the species in this phylogeny.** This NJ tree was extracted from Guinebretière *et al.* 2008 [15] and adapted for the present study. Position of species is mentioned on the right hand of the figure. Also this figure clearly shows the three main branches that lead to group VII (in red), to group I (in pink) and to the remaining groups II to VI (in green). *Bacillus subtilis* was used as an outgroup to root the tree. Strains on the tree were selected strains close to the hypothetical mean organism (HMO) in each phylogenetic Group. **HP**, highly psychrotolerant group; **LP**, low psychrotolerant group; **IM,** intermediate group between psychrotolerance and mesophily; **M**, Mesophilic group; **HM**, highly mesophilic group; **T**, thermotolerant group. Numbers on branch nodes are bootstrap values above 50%.
